# The Synergistic Influence of Polyflavonoids from *Citrus aurantifolia* on Diabetes Treatment and Their Modulation of the PI3K/AKT/FOXO1 Signaling Pathways: Molecular Docking Analyses and In Vivo Investigations

**DOI:** 10.3390/pharmaceutics15092306

**Published:** 2023-09-12

**Authors:** Mohamed A. Hassan, Ghada M. Abd Elmageed, Ibtehal G. El-Qazaz, Doaa S. El-Sayed, Lamia M. El-Samad, Heba M. Abdou

**Affiliations:** 1 Protein Research Department, Genetic Engineering and Biotechnology Research Institute (GEBRI), City of Scientific Research and Technological Applications (SRTA-City), New Borg El-Arab City 21934, Egypt; 2 Department of Zoology, Faculty of Science, Alexandria University, Alexandria 21321, Egypt; dedy862000@yahoo.com (G.M.A.E.); ibtehalgomaa@yahoo.com (I.G.E.-Q.); lamya.moustafa@alexu.edu.eg (L.M.E.-S.); 3 Chemistry Department, Faculty of Science, Alexandria University, Alexandria 21321, Egypt; doaa.saeed@alexu.edu.eg

**Keywords:** diabetes mellitus, diosmin, FOXO1, hesperidin, high fructose/streptozotocin, lemon peel extract-polyflavonoids, PI3K/AKT, AMPK

## Abstract

This study was aimed at probing the modulatory influence of polyflavonoids extracted from *Citrus aurantifolia*, lemon peel extract (LPE-polyflavonoids), on attenuating diabetes mellitus (DM) and its complications. HPLC investigations of the LPE exhibited the incidence of five flavonoids, including diosmin, biochanin A, hesperidin, quercetin, and hesperetin. The in silico impact on ligand-phosphatidylinositol 3-kinase (PI3K) interaction was investigated in terms of polyflavonoid class to explore the non-covalent intakes and binding affinity to the known protein active site. The drug likeness properties and pharmacokinetic parameters of the LPE-polyflavonoids were investigated to assess their bioavailability in relation to Myricetin as a control. Remarkably, the molecular docking studies demonstrated a prominent affinity score of all these agents together with PI3K, implying the potency of the extract to orchestrate PI3K, which is the predominant signal for lessening the level of blood glucose. To verify these findings, in vivo studies were conducted, utilizing diabetic male albino rats treated with LPE-polyflavonoids and other groups treated with hesperidin and diosmin as single flavonoids. Our findings demonstrated that the LPE-polyflavonoids significantly ameliorated the levels of glucose, insulin, glycogen, liver function, carbohydrate metabolizing enzymes, G6Pd, and AGEs compared to the diabetic rats and those exposed to hesperidin and diosmin. Furthermore, the LPE-polyflavonoids regulated the TBARS, GSH, CAT, TNF-α, IL-1β, IL-6, and AFP levels in the pancreatic and hepatic tissues, suggesting their antioxidant and anti-inflammatory properties. In addition, the pancreatic and hepatic GLUT4 and GLUT2 were noticeably increased in addition to the pancreatic p-AKT in the rats administered with the LPE-polyflavonoids compared to the other diabetic rats. Remarkably, the administration of LPE-polyflavonoids upregulated the expression of the pancreatic and hepatic PI3K, AMPK, and FOXO1 genes, emphasizing the efficiency of the LPE in orchestrating all the signaling pathways necessitated to reduce the diabetes mellitus. Notably, the histopathological examinations of the pancreatic and hepatic tissues corroborated the biochemical results. Altogether, our findings accentuated the potential therapeutic role of LPE-polyflavonoids in controlling diabetes mellitus.

## 1. Introduction

Millions of people worldwide are predisposed to a critical threat to their lives due to the prevalence of metabolic diseases, particularly diabetes mellitus (DM), which cannot be understated. DM is a chronic endocrine metabolic disorder identified by augmented blood glucose concentrations and engendered by insulin insufficiency (Type 1 diabetes, T1DM) or insulin resistance (IR), as perceived in type 2 diabetes mellitus (T2DM), and the latter type is prevalent globally [1]. Alarmingly, humans suffering from T2DM are prone to various complications, including cardiovascular diseases, kidney failure, and nerve damage [1,2,3,4]. In T2DM, there are two leading pathophysiological mechanisms, including IR and pancreatic beta cell failure [5].

The pancreas plays a pivotal role in the development of diabetes mellitus, since it orchestrates several homeostatic functions through its exocrine, acinar, and endocrine islet cells [6]. Furthermore, given the property of the pancreas to produce low levels of antioxidant enzymes, pancreatic β-cells are highly prone to oxidative stress (OS) [7]. This OS originates from profuse free radicals, misfolded proteins, and hyperactivity of the endoplasmic reticulum. Consequently, pancreatic β-cells undergo apoptosis on account of this cellular disturbance, instigating pancreatic dysfunction [8]. Furthermore, fructose interferes with glucose metabolism as a result of its specific metabolic fate and lipogenic features. This disturbance provokes IR, dyslipidemia, and hepatic fibrosis, in addition to cardiac and renal malfunctions [9].

It is believed that the impairment of hepatic insulin signaling is predominantly attributed to oxidative damage [10]. Most importantly, the PI3K/AKT signaling pathway is the crucial insulin pathway in glucose metabolism [11]. This signaling pathway is tied up with glucose uptake by the liver, skeletal muscles, and adipose tissues by activating phosphatidylinositol 3-kinase (PI3K), proceeding along the insulin receptor substrates IRS1 and IRS2. The PI3K pathway is the leading modulator of insulin metabolic actions; therefore, a hindrance to this pathway lessens the physiological properties of insulin, which critically provokes IR. Reductions in the glucose utilization and glycogenesis in the liver are the result of these signaling episodes, while hepatic glucose production, glycogenolysis, and triglyceride (TG) buildup are increased [12]. Moreover, multiple signaling pathways are involved in the etiology of T2DM, including adenosine monophosphate-activated protein kinase/sirtuin (AMPK/SIRT1) and adenosine monophosphate-activated protein kinase/mammalian target of rapamycin (AMPK/mTOR) [13].

Importantly, forkhead box-O1 (FOXO1) is one of the forkhead box protein families, which is considerably produced by β-cells; thus, it affects indispensable cellular pathways, such as cell differentiation, metabolism, and cell cycle arrest. It has been strongly evidenced that FOXO1 governs β-cell replication and differentiation [14]. Furthermore, it has been identified as a major influencing protein in T2DM, due to its efficiency in modulating the insulin sensitivity of target tissues [15]. Along with the growth of DM, there has been increasing interest in phenolic and flavonoid compounds from diverse origins to alleviate DM and its detrimental consequences, due to their inherent property of scavenging the overflow of free radical species [16,17], hampering oxidative stress within pancreatic cells [18,19]. Several medicinal plants have introduced alternative products for impeding DM due to their capacity to modify carbohydrate enzymes and adapt glucose homeostasis [20]. Among the plants containing effective flavonoids for different therapies, the citrus plant (*Citrus aurantifolia*), Rutaceae family, is a widespread fruit crop [21]. Grapefruit, lemon, and orange are the principal (dominant) species of citrus, which comprise phenolic components, particularly flavonoids. Citrus peels, which are considered to be worthy by-products, represent the highest flavonoid percentage in whole fruits [22]. It has been argued that lemons could help to reduce the negative consequences of cardiovascular disease, inflammation, tumors, and other malignant diseases [23]. In addition, hesperidin and diosmin are flavonoids abundant in citrus fruits, where the former belongs to the flavanone class and the latter to the flavone glycoside class. Hesperidin is a crucial active component of aged citrus [24]. Both flavonoids possess promising mechanisms as antioxidant, anti-inflammatory, anti-cancer, neuroprotective, lipid-lowering, and glucose-lowering agents [25,26]. In contrast to the available literature discussing the efficacy of hesperidin and diosmin, no research has probed the efficiency of polyflavonoids extracted from lemon peel extract (LPE-polyflavonoids) in alleviating DM. Moreover, no reports have elucidated the potency of LPE-polyflavonoids in mimicking the insulin mechanism in modulating the PI3K/AKT/FOXO1 signaling pathway.

Herein, this study was aimed at identifying the flavonoids in lemon peel extract using high-performance liquid chromatography (HPLC), followed by investigating their binding affinity to PI3K, employing a molecular docking approach. Furthermore, we evaluated the synergistic influence of LPE-polyflavonoids on controlling diabetes mellitus using rats as an animal model, providing extensive evidence concerning their competency as antidiabetic, antioxidant, and anti-inflammatory agents compared to the impact of either hesperidin or diosmin as standard flavonoids from lemons. Furthermore, the potency of the LPE-polyflavonoids in orchestrating PI3K/AKT/FOXO1 signals was assessed. Furthermore, the histological features of pancreatic and liver tissues were surveyed to substantiate their impact on restoring the cellular architectures compared to those organs in the diabetic rat group.

## 2. Materials and Methods

### 2.1. Chemicals and Assay Kits

Streptozotocin (STZ) was provided by Sigma-Aldrich Co., Rockville, MD, USA, while D-Fructose (laevulose) (C_6_H_12_O_6_) was procured from El-Nasr Pharmaceutical Chemicals Co., Cairo, Egypt. Hesperidin 97% (C_28_H_34_O_15_) was purchased from Acros Organics Co., Belgium, WI, USA and diosmin 100% pure extracted from citrus fruit was supplied by Nutrition Greenlife Co., New York, NY, USA.

### 2.2. Preparation of Lemon Peel Extract (LPE)

Lemon plants, *Citrus aurantifolia* (Family: Rutaceae), were collected from the local market and instantly transported to the lab before being identified in terms of their morphological features. The lemon peel extract (LPE) was prepared using a methanol approach following previously reported procedures with minor adaptations [27]. Briefly, lemon peels were sliced into small pieces before being dried at room temperature. Afterward, the dried peels were powdered employing an electric grinder, and about 0.25 g of powder was then extracted for 8 h in 100 mL of methanol under orbital agitation. The extract was then filtered utilizing a polytetrafluoroethylene (PTFE) membrane (0.45 µm pore size). The extraction procedures were repeated three times and the methanol was evaporated at 30 °C under vacuum by the means of a rotary evaporator. The LPE was then stored at 4 °C for further examination.

### 2.3. High-Performance Liquid Chromatography (HPLC) Analysis

To determine the flavonoids in the LPE, 30 mg of LPE powder was incubated in 4 mL of 6 M HCl in a water bath at 100 °C for 15 min with stirring. Following cooling, the residues were filtered and then extracted three times using 15 mL of dichloromethane, followed by a concentration of the extract employing a rotary evaporator under low pressure. Afterward, the extract was dissolved in 10 mL of methanol before being filtered using a filter of 0.45 μm. Next, the sample was injected into an HPLC (Agilent 1260, Santa Clara, CA, USA) connected to a 5C18 column (4.6 mm × 150 mm) and DAD detector. The elution of the flavonoids was conducted utilizing a mobile phase comprising acetonitrile:water:trifluoroacetic acid (20:80:0.01 (*v*/*v*/*v*)) (A) and acetonitrile:trifluoroacetic acid (100:0.1 (*v*/*v*)) (B). The gradient profile was adapted from 0 to 40% B for 0–10 min, 40% B for 10–11 min, and 40 to 100% B for 11–12 min. To restore the initial conditions, 100% mobile phase A was utilized for 6 min at the end of each run. The flow rate was adapted to 0.7 mL/min and the measurement was achieved at 260 nm. The flavonoids were identified with respect to reference flavonoids, and their concentrations were evaluated in the LPE sample.

### 2.4. Molecular Docking Examination of Flavonoids in LPE

Previous studies on medicinal plants have described ligand pocket areas as the leading framework for figuring out the target protein active sites of drugs derived from plants efficiently [28]. Mainly, the docking protocol proceeds via a crucial path for highly effective results [29]. Consequently, the flavonoids detected in the LPE, diosmin (Lig I), biochanin A (Lig II), hesperidin (Lig III), quercetin (Lig IV), and hesperetin (Lig V) were subjected to docking simulation using the Autodock Vina [30,31] software (version 4.2) for its dominant capacity for docking all studied phenolic compounds at the same time under comparable conditions. Furthermore, the chimera software was used for a docking data visualization and analysis [32]. The chosen protein was phosphatidylinositol 3-kinase (PI3K, code ID: 1E90) [33], which was retrieved from the protein data bank website (https://www.rcsb.org/structure/5f0g, accessed on 1 July 2023). Despite the presence of some spectrum ranges of PI3K macromolecule codes, selecting the more in silico-efficient active protein code in our study was considered. The selected target protein was implicated with a reference bioflavonoid-like Myricetin (MYC) lipid-kinase inhibitor. The molecular structures of the main polyphenolic components were downloaded from the PubChem site (https://pubchem.ncbi.nlm.nih.gov/, accessed on 1 July 2023). Prior to commencing the docking process, some key steps were carried out, including the preparation of proteins and ligands in a suitable format. Grid box dimensions, along with the identification of the predicted active sites, were generated. Based on the drug-like control inside the protein pocket, the grid box size was estimated with dimensions of 82 × 92 × 92 Å, with 0.3 Å spacing and grid centers x, y, and z of 10.066, 51.723, and 27.004, respectively, respecting an exhaustiveness of 8 with an energy range equal to 4. The binding affinity mode was considered to be Genetic Algorithm (LGA) [34]. Using the Discovery Studio software (Version 21) (https://www.3ds.com/products-services/biovia/, accessed on 1 July 2023), the results were visualized and the respective figures were exported.

### 2.5. Physicochemical and Pharmacokinetic Protocol

In silico investigations of the drug-like properties of the polyflavonoids and ADMET parameters could be calculated utilizing the open-source online SwissADME tool [35], which deals with the SMILE format. The theoretical interpretations related to the ADMET properties were previously discussed through pkCSM graph-based signatures [36], predicting the small molecules’ efficacy as a promising drug. Furthermore, physicochemical indices can control the Lipinski rules by regulating drug absorption and diffusion.

### 2.6. In Vivo Investigations

Male Wistar albino rats weighing 160–180 g were utilized in the current investigation. The rats were housed at the animal house in the Experimental Animal Center-Medical Research Institute, Alexandria University, Egypt. The animals were sheltered in standard cages following standard laboratory conditions: photoperiod (12/12 h light/dark cycle) and room temperature (22 °C ± 3 °C). The rats were provided with a basal diet and tap water ad libitum. The handling and treatment of the rats were performed according to the ARRIVE guidelines (https://arriveguidelines.org/, accessed on 1 July 2023) and approval for all the animal procedures was obtained from the Institutional Animal Care and Use Committee at Alexandria University, Egypt (approval number: AU04220212302).

#### Experimental Design and Induction of Diabetes

In total, 30 rats were allocated into two groups: Group 1 (control group, 6 rats), which was was administered with distilled water via oral gavage once daily for 4 weeks. Group 2: this group included 24 rats for diabetes induction. Specifically, the 24 rats were provided with 10% fructose in their drinking water for two weeks before being intraperitoneally injected with a single dose of STZ (40 mg/kg body weight) to instigate type 2 diabetes mellitus, as previously reported [37,38]. Following three days of injection, blood was collected from the tail vein to evaluate the concentration of glucose, employing a glucometer, and the rats with fasting blood glucose levels above 220 mg/dL were identified as diabetic rats. Accordingly, the diabetic rats were randomly allocated into 4 sub-groups (n = 6): the Fructose-STZ group, Fructose-STZ + hesperidin (100 mg/kg body weight/day, orally) group, Fructose-STZ + diosmin (100 mg/kg body weight/day, orally) group, and Fructose-STZ + LPE (400 mg/kg body weight/day, orally) group. The hesperidin, diosmin, and LPE-polyflavonoids doses were prepared by dissolving them in distilled water.

### 2.7. Blood Collection and Tissue Preparation

The fasted rats were euthanized by exposure to isoflurane and dissected at the end of the in vivo investigation. Blood samples were collected via cardiac punctures in all the animal groups using a sterile syringe, before being centrifuged at 3000 rpm for 10 min to separate the blood serum. The serum was preserved at −20 °C for further use to evaluate the biochemical parameters. On the other hand, the pancreas and liver were isolated from all the experimental groups and cleaned from blood, before being rinsed with a chilled saline solution. For the biochemical investigations, parts of the pancreatic and hepatic tissues were separately homogenized for 5 min in 2 mL/g tissue of cold buffer (50 mM potassium phosphate pH 7.5, 1 mM EDTA) by the means of a homogenizer (Potter-Elvehjem homogenizer). Afterward, the homogenates were clarified for 15 min at 4000 rpm and 4 °C using a centrifuge (Universal 32 R, Hettich, Tuttlingen, Germany), and the supernatants were preserved at −80 °C. For the molecular analyses, small portions of the pancreas and livers were instantly dried and preserved at −80 °C. For the histological investigations, small slices of the pancreas and liver were fixed in formalin solution (10%).

### 2.8. Biochemical Analysis

The serum glucose was estimated utilizing a commercial assay kit (Bio-diagnostics Co., Cairo, Egypt). The insulin was evaluated utilizing an enzyme-linked immunosorbent assay (ELISA) kit (Cat. No. EMINS, Thermo Fisher Scientific, Waltham, MA, USA), and the glycogen levels were estimated using the Rat Glucagon calorimetric assay kit (Cat. No. ab282931, Abcam Co., Berlin, Germany). Aspartate aminotransferase (AST), alanine transaminase (ALT), alkaline phosphatase (ALP), total protein, and albumin were evaluated in the serum using standard commercial kits from Bio-diagnostics Co., Cairo, Egypt, following the manufacturer’s instructions.

Hepatic hexokinase (HK) was estimated employing an ELISA kit (Cat. No. E4600-100, Biovision Co., Berlin, Germany), hepatic glucokinase activity (GK) was estimated using a kit obtained from MyBioSource Co. (Cat. No. MBS704328, San Diego, CA, USA), hepatic glucose-6-phosphate dehydrogenase (G6Pd) was evaluated utilizing an assay kit purchased from Abcam Co. (Cat. No. ab102529, Berlin, Germany), hepatic Alpha-fetoprotein (AFP) was estimated using a rat AFP ELISA kit (Cat. No. CSB-E08281r; Cusabio Biotech Co., Ltd., Beijing, China), and phospho-AKT was estimated using an ELISA kit obtained from Biovision Co. (Cat. No. K4211-100, Berlin, Germany). All the assays were conducted in six replicates, following the protocols provided by the manufacturer.

#### 2.8.1. Lipid Profile Assay

The total cholesterol was estimated employing a respective kit (Cat. No. MAK043, Sigma-Aldrich Co., Rockville, MD, USA). Furthermore, the triglyceride (TG) level was evaluated utilizing a kit (Cat. No. MAK266, Sigma-Aldrich Co., Rockville, MD, USA). HDL-cholesterol was estimated following the approach proposed by Lopes-Virella et al. [39], while LDL-cholesterol was assessed utilizing a kit supplied by Sigma-Aldrich Co. (Cat. No. MAK045, Rockville, MD, USA).

#### 2.8.2. Assessment of Oxidative Stress Biomarkers

To evaluate the lipid peroxidation in the tissue homogenates, thiobarbituric acid reactive substance (TBARS) was estimated, as described earlier [40], while glutathione (GSH) was assessed spectrophotometrically following the protocol reported by Jollow et al. [41]. The activities of superoxide dismutase (SOD) and catalase (CAT) were estimated following the procedures of Nishikimi et al. and Abei et al., respectively [42,43]. Additionally, the advanced glycation end-products’ (AGEs) concentrations were estimated utilizing the OxiSelect™ AGE Competitive ELISA Kit (Cell Biolabs, San Diego, CA, USA). All the investigations were replicated six times.

#### 2.8.3. Evaluation of Inflammatory Biomarkers

To quantify the tumor necrosis factor alpha (TNF-α), interleukin-6 (IL-6), and nuclear factor Kappa (NF-κB), ELISA kits obtained from MyBioSource Co., San Diego, CA, USA were utilized (Cat. No. MBS2507393, Cat. No. MBS726707, and Cat. No. MBS453975, respectively). Additionally, interleukin-1β (IL-1β) was assessed adopting a respective ELISA kit (Cat. No. BMS630, Thermo Fisher Scientific, Waltham, MA, USA).

#### 2.8.4. Glucose Transporter 2 (GLUT2) and GLUT4 and p-AKT

The concentrations of glucose transporter 2 (GLUT2) and glucose transporter 4 (GLUT4) were quantified in the liver and pancreatic tissues using sandwich ELISA kits from MyBioSource Co., San Diego, CA, USA (Cat. No. MBS2025604 and Cat. No. MBS2023267, respectively). Using a similar approach, phospho-AKT (p-AKT) was evaluated in the pancreas utilizing a phospho-AKT ELISA kit (Cat. No. K4211-100, Biovision Co., Berlin, Germany).

#### 2.8.5. Quantitative Real-Time PCR (qRT-PCR) Analyses

The relative mRNA expression levels of the phosphatidylinositol 3-kinase (PI3K), adenosine monophosphate-activated protein kinase (AMPK), and forkhead box-O1 (FOXO1) genes were assessed using qRT-PCR. The RNA was obtained from the pancreas and liver utilizing Trizol Reagent (Thermo Fisher Scientific, Waltham, MA, USA), following the manufacturer’s protocol. Subsequently, the qRT-PCR was conducted employing the Rotor-Gene SYBR Green RT-PCR Kit (Qiagen, Montgomery, MD, USA), following the protocols supplied by the manufacturer. Briefly, 2x Rotor-Gene SYBR Green RT-PCR Master Mix, template RNA (≤100 ng/reaction), primers, and RNase-free water were mixed and put on ice. The cDNA synthesis was carried out at 55 °C for 10 min and then at 95 °C for 5 min for RNase deactivation. Afterward, the qRT-PCR was initiated, and the temperature and time for the annealing step were optimized for each reaction according to the primer used in the PCR reaction. The sequences of primers applied for the qRT-PCR are shown in Table 1. The fold difference was computed using the Equation (2) −ΔΔct relative to β-actin, as reported earlier [44,45].

### 2.9. Histopathological Examination

The pancreatic and liver tissues were fixed by immersing them in 10% neutral-buffered formalin for 48 h. Next, the tissues were dehydrated in increasing concentrations of ethanol for 15 min for each concentration, before being embedded in paraffin wax. Subsequently, they were sectioned (4 μm thickness), prior to being stained with Hematoxylin and Eosin (H&E). The H&E sections were surveyed by the means of a light microscope (Olympus CX31, Tokyo, Japan).

### 2.10. Statistical Analysis

The statistical analysis was accomplished by the means of Statistical Product and Service Solutions (SPSS), version 25, produced by IBM Software, Inc., Chicago, IL, USA. The data were normalized adopting a Kolmogorov–Smirnov test. Multiple comparisons of the data were performed using a one-way analysis of variance (ANOVA) and Tukey’s post hoc test. All the data are shown as means ± standard deviation (SD). Differences were considered significant at *p* ≤ 0.05.

## 3. Results

### 3.1. HPLC Study on Flavonoid Profiles

**Figure 1:**
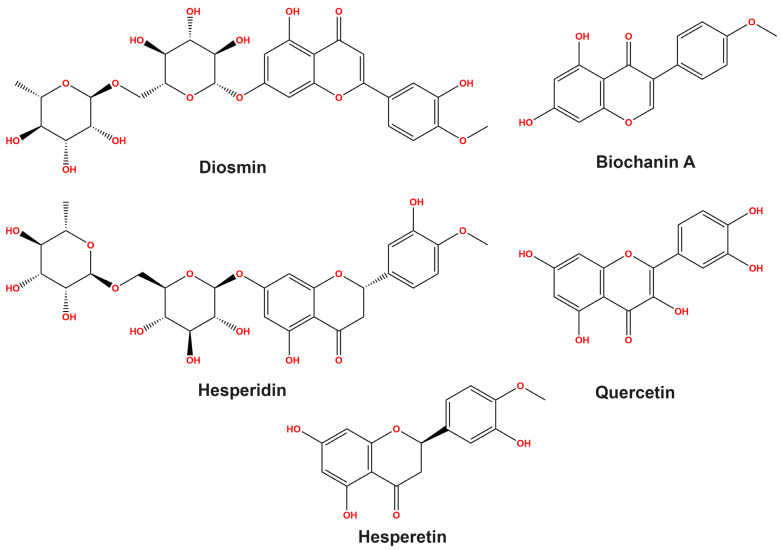
Chemical structures of flavonoid compounds identified in LPE.

The HPLC analysis of the flavonoids in the LPE exhibited the emergence of five flavonoid compounds, including diosmin, biochanin A, hesperidin, quercetin, and hesperetin at different retention times with concentrations of 62.75, 62.35, 59.18, 28.32, and 9.31 mg/kg, respectively, as depicted in Table 2. Furthermore, Figure 1 delineates the chemical structures of the flavonoids identified in the LPE, in accordance with the reference flavonoids.

### 3.2. Potential Interaction of the PI3K Protein with LPE-Polyflavonoids

**Figure 2:**
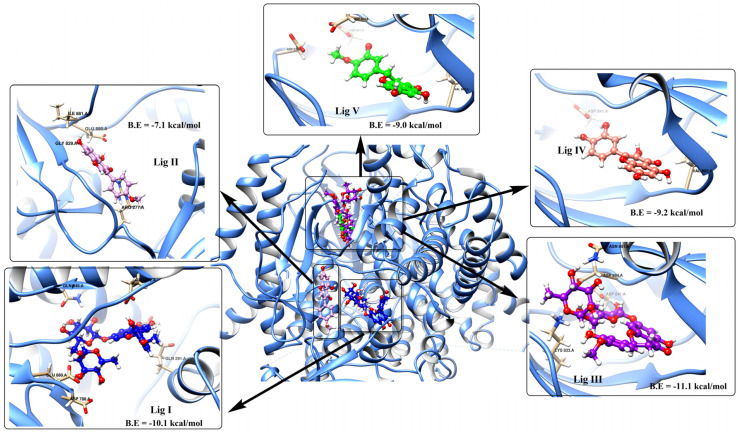
Docking score of the LPE-polyflavonoids ligands detected in the LPE with target protein 1E90, involving the best score value. B.E indicates the value of binding energy.

**Figure 3:**
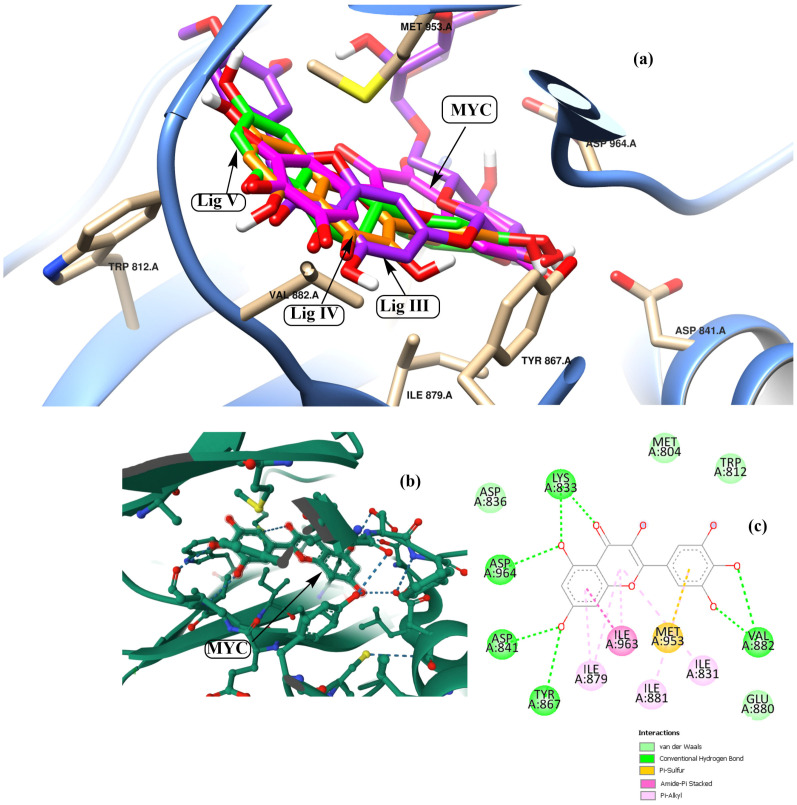
Graphical map describes Lig III, Lig IV, and Lig V superimposed with MYC control (pink color code) in the drug-potent active site (**a**), snapshot [https://www.rcsb.org/3d-view/1E90?preset=ligandInteraction&label_asym_id=B] (accessed on 1 September 2023) of the reported PI3K active sites complex with MYC (**b**), and (**c**) 2D colored interaction map related to MYC active pocket.

The ligand–protein complex binding mode is best described by a docking simulation analysis with minimum energy generation [46,47]. Proper binding requires protein active site detection, which was depicted for obtaining the site-best score. Figure 2 reveals the results obtained from the docking of the five studied ligands aligned with the best binding energy pose and incorporated with the reference MYC bioactive ligand. The visualized map arranged Lig III (hesperidin) as the best ligand, with a binding energy of −11.1 kcal/mol, followed by Lig I (diosmin), with a value of −10.1 kcal/mol. The score parameter values for Lig IV, Lig V, and Lig II were −9.2, −9.0, and −7.1 kcal/mol, respectively. In more detail, the active sites of PI3K bound to each ligand were mostly different in the binding mode. Based on the sign of the MYC control, Figure 3 discriminates the reference point of the interaction between the control and the binding amino acids of the lipid kinase PI3K. The MYC active site includes some important favorable interactions, particularly conventional H-bonds with the key residues LYS^833^, ASP^841^, TYR^867^, VAL^882^, and ASP^964^. Other non-covalent interactions were predicted, such as π-sulfur, π-amide-stacked, and π-alkyl types. Furthermore, the studied polyflavonoids were directed to the same potent pocket, demonstrating significant activity towards PI3K inhibition. It could be perceived from the docking process that Lig III, Lig IV, and Lig V were superimposed on MYS inhibitors, predicting similar behavior in the inhibition process. Additionally, it was observed that Lig III worked with most of the active amino acids in common with the bound-control crystallographic complex.

**Figure 4:**
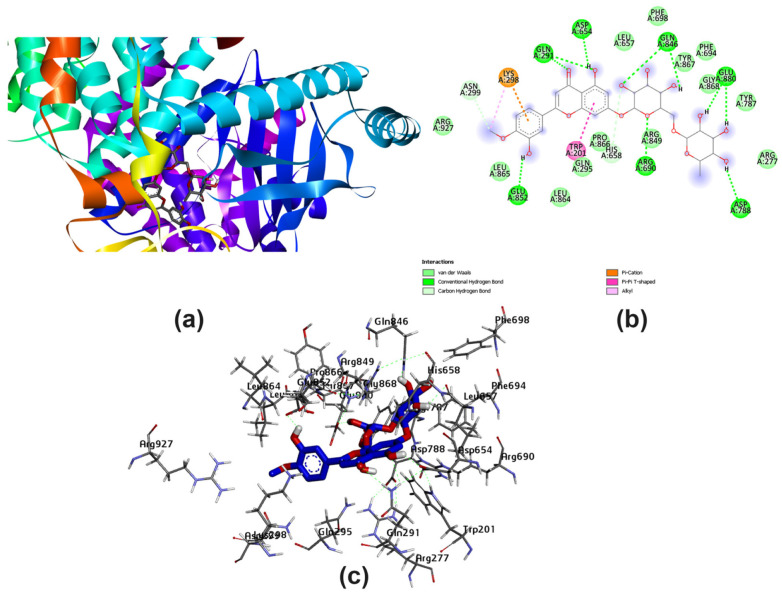
Docking analysis of Lig I with best score pattern: (**a**) 3D map of ligand–receptor docked complex with rainbow ribbon color code, (**b**) 2D interaction map with several 1E90-amino acids, and (**c**) 3D-generated ligand–protein complex.

**Figure 5:**
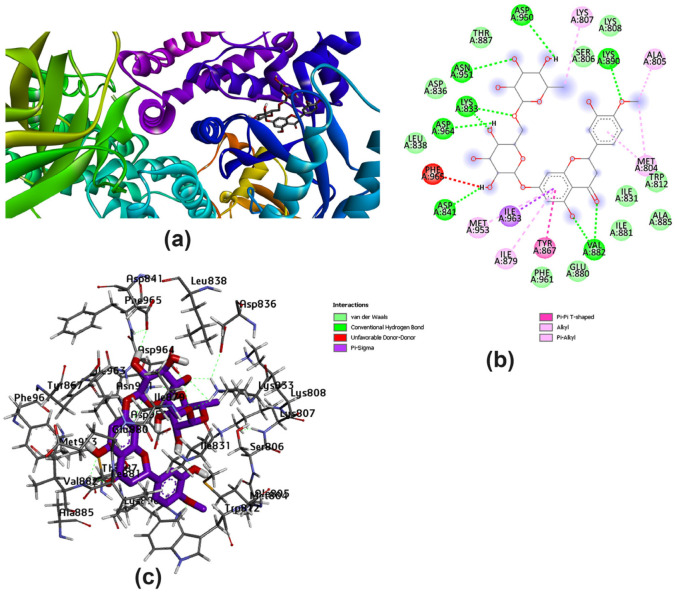
Docking analysis of Lig III with best score pattern: (**a**) 3D map of ligand–receptor docked complex with rainbow ribbon color code, (**b**) 2D interaction map with several 1E90-amino acids, and (**c**) 3D-generated ligand–protein complex.

**Figure 6:**
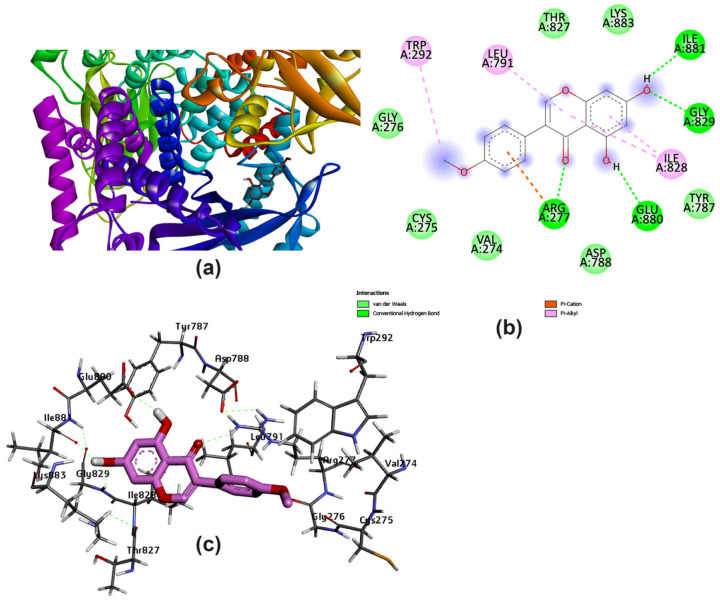
Docking analysis of Lig II with best score pattern: (**a**) 3D map of ligand–receptor docked complex with rainbow ribbon color code, (**b**) 2D interaction map with several 1E90-amino acids, and (**c**) 3D-generated ligand–protein complex.

**Figure 7:**
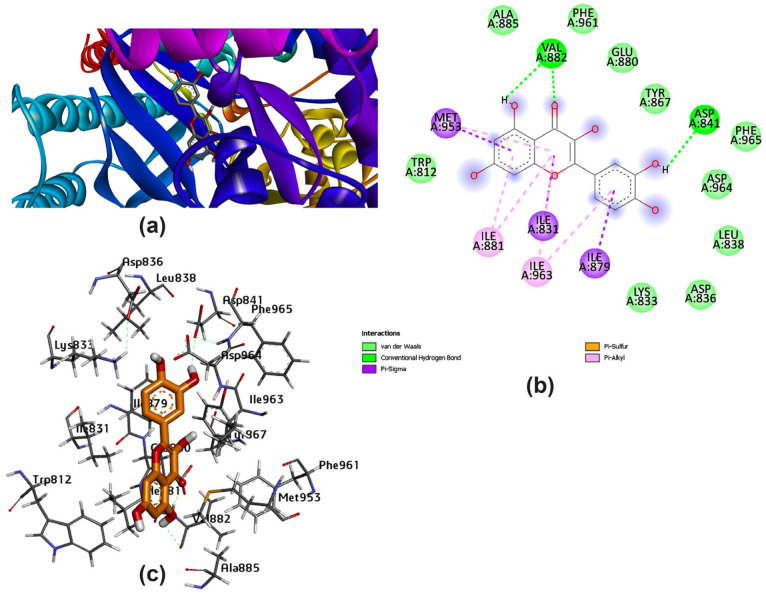
Docking analysis of Lig IV with best score pattern: (**a**) 3D map of ligand–receptor docked complex with rainbow ribbon color code, (**b**) 2D interaction map with several 1E90-amino acids, and (**c**) 3D-generated ligand–protein complex.

**Figure 8:**
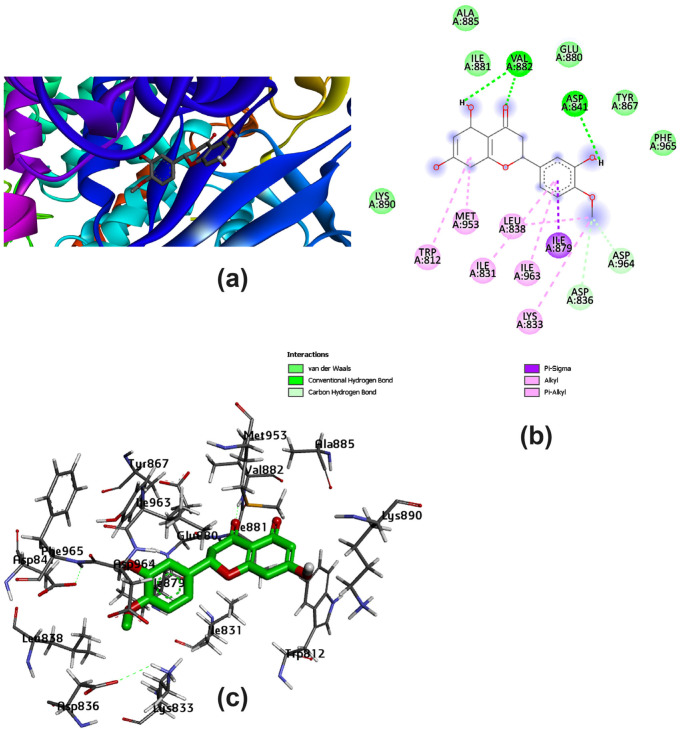
Docking analysis of Lig V with best score pattern: (**a**) 3D map of ligand–receptor docked complex with rainbow ribbon color code, (**b**) 2D interaction map with several 1E90-amino acids, and (**c**) 3D-generated ligand–protein complex.

Several amino acids that interacted with Lig I and Lig III are delineated in Figure 4 and Figure 5, demonstrating a variety of interaction types, particularly conventional and carbon–hydrogen bonds, where these mainly assisted with further complex stabilization. The majority preferred Lig I and Lig III as potent inhibitors, due to the large number of functional groups in the molecule compared to the other docked ligands. Figure 6, Figure 7 and Figure 8 represent the docking analyses of Lig II, Lig IV, and Lig V, which revealed lower scores due to the presence of a smaller number of protein–site interactions.

### 3.3. Pharmacokinetic Parameters and Drug-Likeness Prediction

The Lipinski rules can assess the drug properties’ validity in the studied compounds based on five determinants: a molecular weight of < 500 Da and a high lipophilic property, including a partition coefficient LogP value of < 5, H-bond donors < 5, H-bond acceptors < 10, and a molar refractivity value occurring between 40 and 130. Achieving more than two of these determinants can allow for the studied compound to have drug-like properties [48]. Other filters, such as Ghose, Veber, and Egan, for the studied polyflavonoids substantially help in identifying the bioavailability score [49], which was more significant in Lig II, Lig IV, and Lig V. Table 3 displays the pharmacokinetic parameters and drug-likeness properties elucidated for the studied compounds.

Pharmacokinetic properties also deal with the availability of a predicted drug for partitioning in gastrointestinal cells with high proportions, such as Lig II, Lig IV, and Lig V, and low proportions such Lig I and Lig III, which resemble the control effect. The blood–brain barrier (BBB) parameter is an indicator of the proportional distribution of the studied molecules, where the current flavones are undistributed in human brain blood. Metabolism factors can estimate a compound’s tendency to inhibit five isoenzymes (CYP1A2, CYP2C19, CYP2C9, CYP2D6, and CYP3A4) related to cytochrome P450 enzymes. This property is a crucial contributor to toxicity and other medication side effects [50]. In this study, it was found that Lig III exhibited no function as an inhibitor for the five isoenzymes compared to the other candidates.

### 3.4. Impact of LPE-Polyflavonoids on Serum Glucose, Insulin, Glycogen, and Liver Enzymes

**Figure 9:**
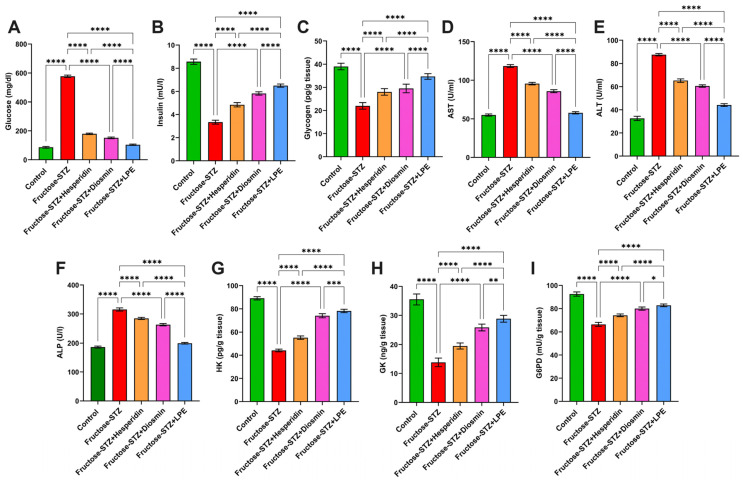
Evaluation of (**A**) glucose, (**B**) insulin, and (**C**) glycogen in the serum of different animal groups. Analysis of liver function enzymes, including (**D**) AST, (**E**) ALT, and (**F**) ALP in the serum of experimental animal groups. Estimation of hepatic carbohydrate-metabolizing enzymes, including (**G**) HK, (**H**) GK, and (**I**) G6PD in animal groups. The results are depicted as mean ± SD when n = 6, which were analyzed following one-way ANOVA associated with multiple comparisons between different groups adopting Tukey’s test (**** *p* < 0.0001, *** *p* < 0.001, ** *p* < 0.01, and * *p* < 0.05). Abbreviations: AST: Aspartate aminotransferase; ALT: alanine transaminase; ALP: alkaline phosphatase; HK: hepatic hexokinase; GK: glucokinase; and G6Pd: glucose-6-phosphate dehydrogenase.

The level of glucose and activities of AST, ALT, and ALP were substantially augmented in the Fructose-STZ rats in comparison to the control rats, as portrayed in Figure 9A,D–F. Moreover, it was apparent from the data in Figure 9B,C that the levels of insulin and glycogen were noticeably diminished in the Fructose-STZ group in comparison to the control group. In contrast, the rats administered with hesperidin and diosmin demonstrated remarkable enhancements in terms of glucose, insulin, glycogen, and the performance of their liver enzymes. Interestingly, the rats administered with the LPE manifested significant enhancements in relation to these parameters compared to all the other treated rats, implying a synergistic influence of flavonoids in restoring cellular functions.

### 3.5. Influence of LPE-Polyflavonoids on Hepatic Carbohydrate-Metabolizing Enzyme Activities

It can be observed from Figure 9G–I that the activities of GK, HK, and G6Pd in the Fructose-STZ group markedly dwindled (*p* ≤ 0.05) in comparison to those of the control animals. On the contrary, the treatment with hesperidin and diosmin resulted in remarkable growth in the levels of GK, HK, and G6Pd. Moreover, the rats exposed to the LPE showed significant increases (*p* ≤ 0.05) in these enzymes, more so than those treated with either hesperidin or diosmin, which supports the previous findings.

### 3.6. Effect of LPE-Polyflavonoids on Total Protein and Albumin Levels

**Figure 10:**
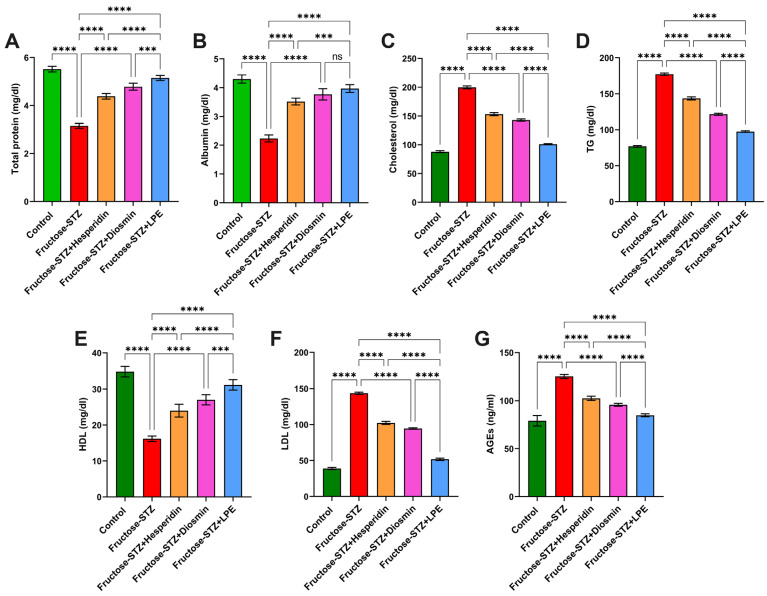
Assessment of (**A**) total protein and (**B**) albumin in the serum of different animal groups. Changes in the serum lipid profiles of experimental groups involving (**C**) Total cholesterol, (**D**) TG, (**E**) LDL-C, and (**F**) LDL of animal groups. Estimation of (**G**) AGEs levels in animal groups. The results are exhibited as mean ± SD when n = 6, which were analyzed following one-way ANOVA associated with multiple comparisons between different groups adopting Tukey’s test (**** *p* < 0.0001, *** *p* < 0.001, and ns alludes to non-significant differences). Abbreviations: TG: triglyceride (TG); HDL-cholesterol; LDL-cholesterol; and AGEs: advanced glycation end-products.

The diabetic rats treated with Fructose-STZ showed noticeable diminutions in their total protein and albumin in relation to the control rats, as displayed in Figure 10A,B. In contrast, the rats treated only with hesperidin or diosmin demonstrated marked increases in their concentrations of total protein and albumin. Importantly, the treatment with the LPE caused further enhancements in both factors, with a significant difference (*p* ≤ 0.05) in comparison to all the diabetic rats. Altogether, these results corroborate that the treatment with the LPE, including the polyflavonoids, had a vital influence on the treatment of diabetes.

### 3.7. Influence of LPE-Polyflavonoids on Serum Lipid Profile and AGEs

Figure 10C–F exhibit that there were obvious trends of remarkable increases in the concentrations of serum total cholesterol, TG, and LDL-C in the Fructose-STZ rats, associated with a substantial decrease in comparison to the control rats. With regard to AGEs, as a fundamental biomarker for proteins and lipids glycated as a consequence of reactions with sugars, the diabetic rats in the Fructose-STZ group evinced a considerable escalation in their AGEs compared to the untreated rats, as presented in Figure 10G. On the contrary, the administration of hesperidin or diosmin to the diabetic rats exposed ameliorative influences in relation to the lipid profiles, associated with a noticeable reduction in AGEs. Most notably, the LPE-treated rats demonstrated higher enhancements in their lipid parameters and AGEs compared to those treated only with one of the flavonoid compounds, reflecting the key role of polyflavonoids in the LPE for managing diabetes and its further deleterious effects.

### 3.8. Effect of LPE-Polyflavonoids on Oxidative Stress Biomarkers in Pancreatic and Liver Tissues

**Figure 11:**
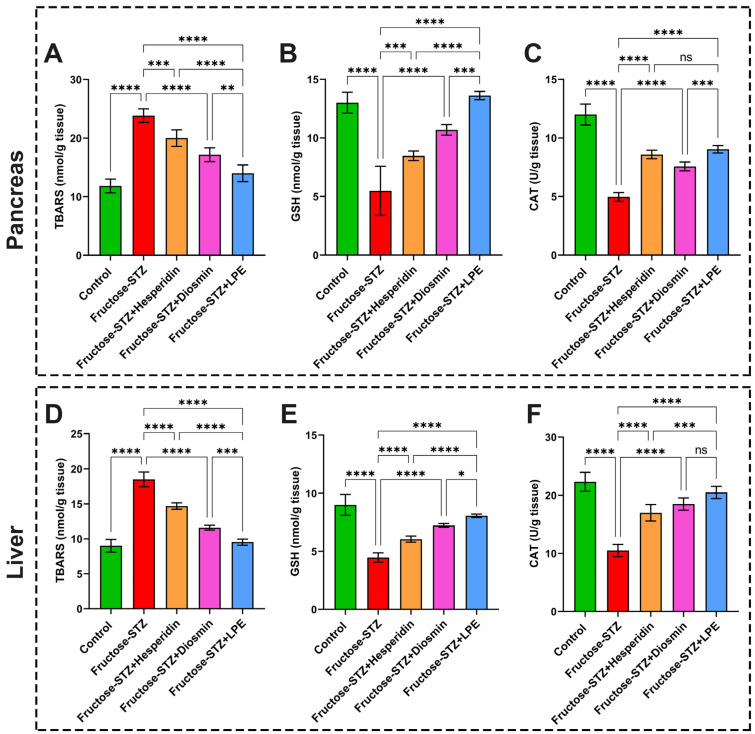
Evaluation of oxidative stress biomarkers and catalase in pancreatic and hepatic tissues of different animal groups. (**A**,**D**) TBARS levels in pancreatic and hepatic tissues, respectively, to appraise the lipid peroxidation. (**B**,**E**) GSH concentrations in pancreatic and hepatic tissues, respectively. (**C**,**F**) CAT activity in pancreatic and hepatic tissues, respectively. The results are shown as mean ± SD when n = 6, which were analyzed following one-way ANOVA associated with multiple comparisons between different groups adopting Tukey’s test (**** *p* < 0.0001, *** *p* < 0.001, ** *p* < 0.01, * *p* < 0.05, and ns refers to non-significant differences). Abbreviations: TBARS: thiobarbituric acid reactive substance; GSH: glutathione; and CAT: catalase activity.

Concerning the oxidative stress biomarkers in the Fructose-STZ group, the results exhibited noticeable disorders in the TBARS, GSH, and CAT levels. Precisely, the level of lipid peroxidation was markedly amplified through a determination of the TBARS content in both the pancreatic and liver tissues, linked with significant drops in the GSH and CAT levels in relation to the control rats, as shown in Figure 11A–F. Conversely, the application of hesperidin or diosmin counteracted these detrimental impacts, indicating their efficiency in treating diabetes. Remarkably, the LPE-polyflavonoids alleviated the oxidative stress levels in the liver and pancreas, with significant differences in relation to one of the flavonoids, suggesting a synergistic influence of the five flavonoids, which impart the LPE with intrinsic antioxidant attributes.

### 3.9. Impact of LPE-Polyflavonoids on Liver and Pancreas Inflammatory Markers, NF-κB, and AFP

**Figure 12:**
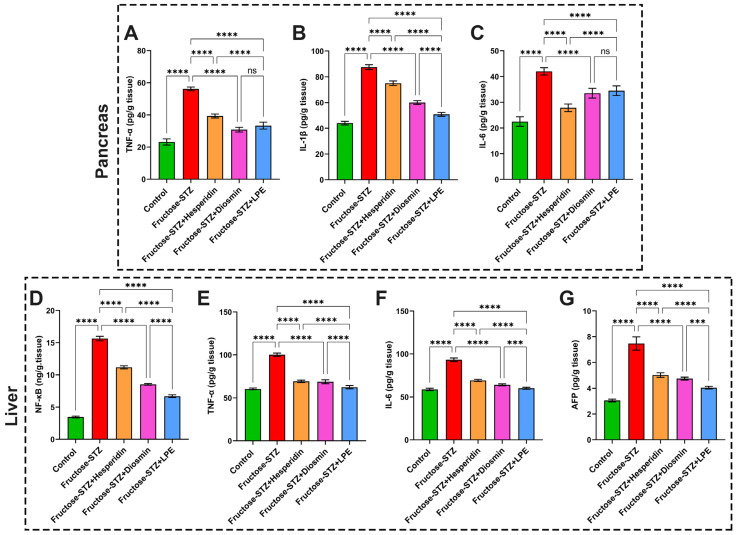
Estimation of inflammatory biomarkers in pancreatic and liver tissues in addition to hepatic Alpha-fetoprotein (AFP) from different rat groups. (**A**,**E**) TNF-α concentrations in pancreatic and hepatic tissues, respectively. (**B**) IL-1β in pancreatic homogenates. (**C**,**F**) IL-6 levels in pancreatic and hepatic tissues, respectively. (**D**) NF-κB and (**G**) AFP in hepatic tissues of animal groups. The findings are exhibited as mean ± SD when n = 6, which were analyzed following one-way ANOVA associated with multiple comparisons between different groups adopting Tukey’s test (**** *p* < 0.0001, *** *p* < 0.001, and ns points to a non-significant difference). Abbreviations: TNF- α: tumor necrosis factor alpha; IL-1β: interleukin-1β; IL-6: interleukin-6; NF-κB: nuclear factor kappa B; and AFP: Alpha-fetoprotein.

The results of the inflammatory markers demonstrated significant augmentations in the TNF-α, IL-1β, and IL-6 in the pancreatic tissues obtained from the Fructose-STZ-treated group in relation to the control animals, as delineated in Figure 12A–C. Moreover, Figure 12D–G illustrate that the concentrations of TNF-α, IL-6, NF-κB, and AFP were markedly heightened in the hepatic tissues of the diabetic animals. In contrast to these results, the rats treated with hesperidin or diosmin showed significant mitigations of inflammatory marker production in both their pancreatic and liver tissues, in addition to a discernible reduction in the NF-κB and AFP levels in their hepatic tissues. Furthermore, the rats administered with the LPE revealed a noticeable modulation of their inflammatory marker levels alongside NF-κB and AFP, better than that in all the diabetic rat groups. These findings substantiate the crucial contribution of the LPE to controlling diabetes and verify the previous results.

### 3.10. Effect of LPE-Polyflavonoids on GLUT4 and GLUT2 in Pancreas and Liver Tissues

**Figure 13:**
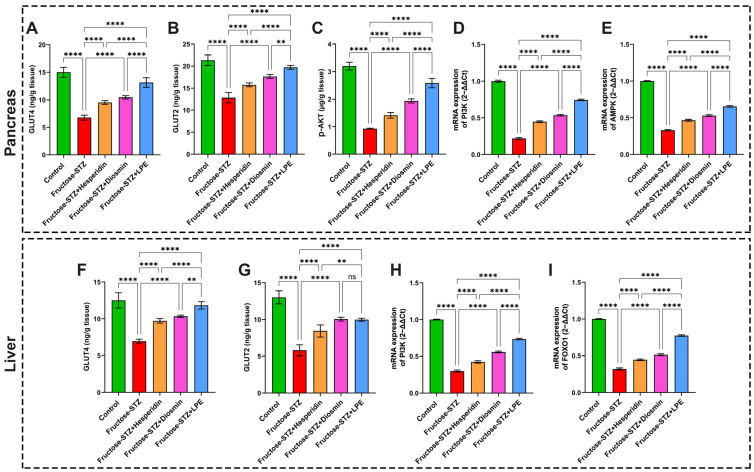
Determination of (A) and (F) GLUT4 levels in pancreatic and hepatic tissues, respectively. (**B**,**G**) GLUT2 concentrations in pancreatic and hepatic tissues, respectively. (**C**) p-AKT concentration in pancreatic tissues of different rat groups. (**D**,**H**) mRNA expression of PI3K in pancreatic and hepatic tissues, respectively. (**E**) mRNA expression of AMPK in the pancreas and (**I**) mRNA expression of FOXO1 in liver. The results are shown as mean ± SD when n = 6, which were analyzed following one-way ANOVA associated with multiple comparisons between different groups adopting Tukey’s test (**** *p* < 0.0001, ** *p* < 0.001, and ns indicates a non-significant difference). Abbreviations: GLUT4: Glucose transporter 4; GLUT2: Glucose transporter 2; p-AKT: phospho-Akt or protein kinase B; PI3K: phosphatidylinositol 3-kinase; AMPK: adenosine monophosphate-activated protein kinase; and FOXO1: forkhead box-O.

It was evident from the GLUT4 and GLUT2 results that the levels of both parameters in the pancreases and livers obtained from the Fructose-STZ-treated rats were remarkably lower than those observed in the control group, as presented in Figure 13A,B,F,G. Compared to the Fructose-STZ group, the treatments with hesperidin, diosmin, and the LPE gave rise to notable increases (*p* ≤ 0.05) in GLUT4 and GLUT2, with the highest levels seen in the LPE group.

### 3.11. Effect of LPE-Polyflavonoids on p-AKT in the Pancreas and Gene Expressions of PI3K, AMPK, and FOXO1 in Pancreatic and Hepatic Tissues

In the Fructose-STZ-treated group, it was distinct that diabetes instigated a significant diminution (*p* ≤ 0.05) in the pancreatic p-AKT, alongside notable downregulations of the pancreatic PI3K and AMPK signals compared to the control rats, as illustrated in Figure 13C–E. Additionally, it can be perceived from the results in Figure 13H,I that the gene expressions of hepatic PI3K and FOXO1 were markedly reduced in the untreated diabetic rats. Nevertheless, the administration of hesperidin, diosmin, and the LPE to the diabetic rats substantially promoted the level of the p-AKT signal and upregulated the mRNA expressions of PI3K and AMPK in the pancreas tissues and PI3K and FOXO1 in the liver tissues. Moreover, it was discernible that the treatment with the LPE-polyflavonoids markedly promoted the expressions of various diabetic modulators, higher than those treated with one flavonoid agent. Collectively, these findings emphasize the pivotal functions of flavonoids, including LPE-polyflavonoids, as countermeasures to oxidative stress and other complications derived from diabetes by orchestrating the expressions and functions of PI3K, p-AKT, and FOXO1.

### 3.12. Histopathological Analysis of Pancreatic and Liver Tissues

**Figure 14:**
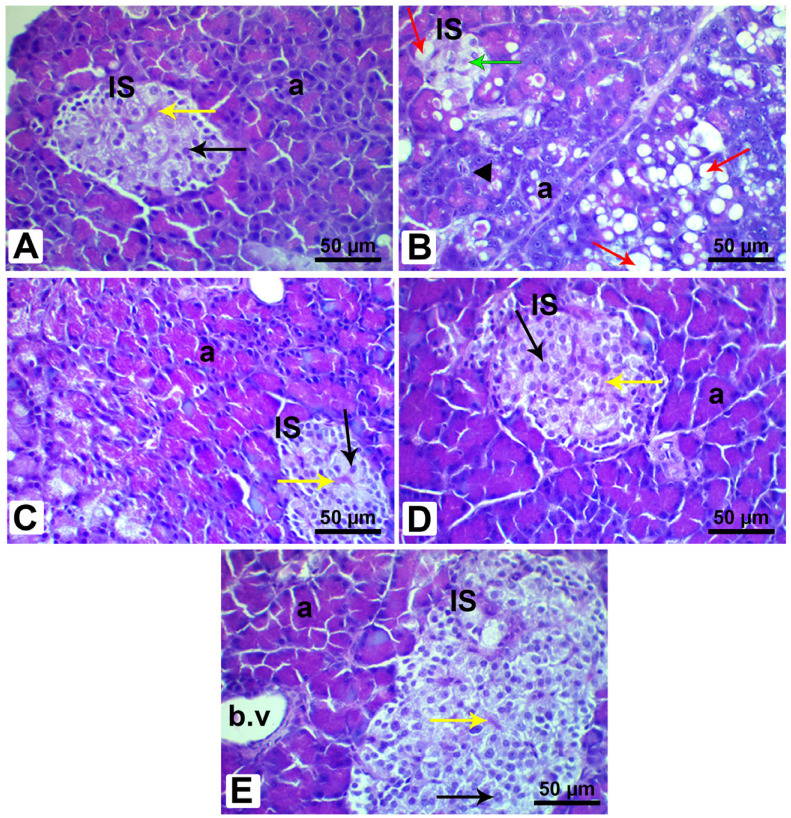
Photomicrographs of pancreatic sections from male rats stained with H&E. (**A**) Section of the control pancreas, exhibiting normal pancreatic structure, typical pancreatic acini with pyramidal-shaped cells and rounded nuclei (a), regular pale-stained islet of Langerhans (IS) with typical ß cells (black arrow) and a blood vessel (yellow arrow). (**B**) Pancreas from Fructose-STZ-treated rats, revealing a shrunken, distorted islet of Langerhans with loss of ß cells (green arrow), emergence of vacuolated cytoplasm (red arrow), acinar cells with pyknotic nuclei (black arrowhead), and incidence of fat infiltration (red arrow). (**C**) Pancreatic sections of diabetic rats treated only with hesperidin, (**D**) pancreatic sections of diabetic rats treated only with diosmin, and (**E**) pancreatic sections of diabetic rats treated with LPE-polyflavonoids, demonstrating restoration of the regular pancreatic architecture, with particular improvement in rats treated with LPE-polyflavonoids, including typical pancreatic acini (a), a regular pale-stained islet of Langerhans (IS) with regular ß cells (black arrow) and blood vessels (yellow arrow).

**Figure 15:**
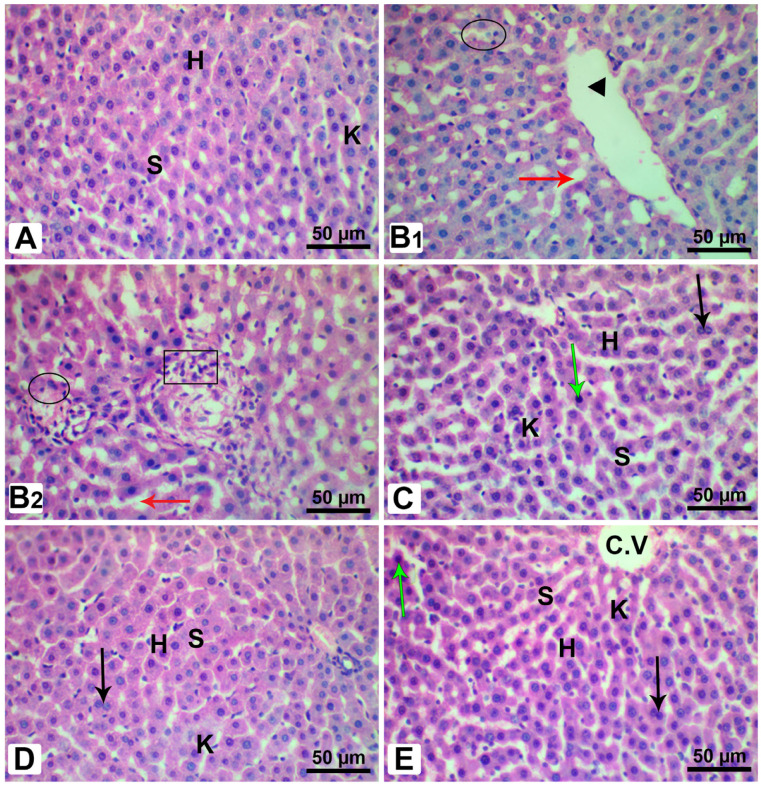
Photomicrographs of hepatic sections from male rats stained with H&E. (**A**) Section of the control liver from control rats, revealing typical histological structure, including the regular pattern of hepatocytes (H), typical blood sinusoids (S), and Kupffer cells (K). (**B1**,**B2**) Liver sections from Fructose-STZ-treated rats, showing dilation of the central vein (head arrow) and dilation of the blood sinusoids (red arrow), pyknotic nuclei (black circle), and accumulation of inflammatory cells among hepatocytes (black square). (**C**) Liver sections of diabetic rats treated only with hesperidin, (**D**) liver sections of diabetic rats administered only with diosmin, and (**E**) liver sections of diabetic rats treated with LPE-polyflavonoids, demonstrating ameliorations in the histological structures associated with the appearance of the central vein (C.V.), regular organization of hepatocytes (H), typical blood sinusoids (S), and Kupffer cells (K), which could be perceived in the control rats, except for the emergence of few pyknotic nuclei (green arrow) and binucleated cells (black arrow). Compared to all groups of diabetic rats, it could be discerned that there was substantial restoration of liver architecture in rats treated with LPE-polyflavonoids.

To provide further support for the previous findings, histological sections of the pancreases and livers of the rats in all the experimental groups were investigated, as presented in Figure 14 and Figure 15. It can be perceived from the H&E sections of the pancreatic tissues dissected from the control rats that the pancreas exposed a regular cellular architecture associated with typical large Langerhans islets and normal pyramidal-shaped cells “pancreatic acini”, as depicted in Figure 14A. In contrast, Figure 14B illustrates that the untreated diabetic rats demonstrated aberrations in their cellular structures of Langerhans islets, including a retraction and distortion of the islets correlated with a lack of B cells, cytoplasmic vacuolation, and the pyknotic nuclei of several islet cells. Prominently, the diabetic rats treated with hesperidin, diosmin, and the LPE restored the major pancreatic architecture close to that of the control group, as portrayed in Figure 14C–E.

Considering the histopathological inspection of the livers, it is evident from Figure 15A that the control rats manifested a standard hepatic architecture, involving sinusoids and the central vein. On the other hand, the hepatic tissues from the Fructose-STZ rats revealed a dilation of the central vein and blood sinuses and the necrosis of hepatocytes with nuclear pyknosis, in addition to extensive inflammatory cells among the hepatocytes, as shown in Figure 15B1,B2. In contrast to these findings, the rats administered with hesperidin, diosmin, and the LPE exhibited a reinstatement of histological features, except for the emergence of a few pyknotic nuclei and binucleated cells, as represented in Figure 15C–E.

## 4. Discussion

It is imperative to implement comprehensive strategies that focus on the impediments, early detection, and effective management of T2DM and its deleterious consequences. Among the various therapies for hampering diabetes and its complications, phenolic and flavonoid compounds have been receiving profuse attention due to their antioxidant and anti-diabetic properties [18,51,52]. In our study, we investigated the efficiency of LPE-polyflavonoids in alleviating DM and enhancing the pancreatic and liver functions by regulating the expressions of PI3K, p-AKT, and FOXO1 as key modulators for controlling DM. The results manifested a prevalence of diosmin, biochanin A, hesperidin, quercetin, and hesperetin in the LPE in different amounts.

Considering the docking simulation of the designed polyflavonoids with the lipid-modifying enzyme PI3K, it was illustrated that most MYS-binding residues were active with the studied ligands, especially for Lig III. The genetic algorithm docking proceeded via a binding score generation for the designed polyflavonoids to conclude the binding energy values. A lower binding energy score initiates a higher affinity for inhibition. Accordingly, Lig III showed a lower energy value (−11.1 kcal/mol). Furthermore, the predicted docked ligand-active site for Lig III, Lig IV, and Lig V was congruent with the key site of the reference MYC inhibitor. To analyze the 2D map interactions for all the studied ligands and compare the results with MYC, it was projected that Lig III and Lig IV interacted with most key residues of the control. Such residues, which interact with some non-covalent interactions, were LYS^833^, ASP^964^, ASP^841^, VAL^882^, MET^953^, and ILE^879^. Assuming the importance of the pharmacokinetic and drug-likeness properties [53], ADME parameters were provided to find that the polyflavonoids had drug-like properties in relation to the MYC inhibitors. Despite the bioavailability prediction, with a score of 0.55 for Lig III, the Lipinski rule detected three unachievable factors of the total five indices. Molecular weight (Mwt = 610.56 Da) is the main factor involved in evaluating drug-like properties. Furthermore, the pan-assay interferences (PAINS) reported no alert for most of the studied ligands. This factor was detected for Lig IV and the control in the same examination [54]. Previous investigations have reported that the binding of phenol and flavonoid compounds to proteins adapts their conformational structure, engendering alterations in their intrinsic protein properties and functions, such as solubility and isoelectric point [19]. Altogether, the molecular docking studies highlighted the potency of all five flavonoid agents in binding to the p-AKT protein, which mimics the insulin mechanism to inhibit the augmentation of glucose in the blood.

To emphasize the prospective application and evaluation of the extent to which the LPE-polyflavonoids thwarted DM, in vivo investigations, alongside extensive evaluations of various biochemical factors, were conducted. The in vivo results indicated a significant elevation in the level of glucose, with a reduction in the insulin level in the Fructose-STZ group, which could have been related to increased oxidative stress as a result of the progression of diabetes, followed by an oxidative degradation of glycated proteins [55]. Furthermore, it was believed that β-cells survived the STZ toxicity and became dysfunctional, resulting in high blood glucose levels and a subsequent toxicity of glucose [56]. Moreover, poor GLUT2-mediated glucose sensing is a key factor in the emergence of glucose intolerance and a manifestation of β-cell malfunction [57].

Our findings evinced that the treatment of the diabetic rats with the LPE-polyflavonoids led to improvements in their glucose and insulin levels, greater than those in the rats administered with a single agent of hesperidin and diosmin. It was identified that diosmin and hesperidin possess anti-diabetic properties, which are exerted primarily through glucose metabolism regulation [58,59]. Additionally, diosmin nanoparticle therapy modulated the glucose and insulin levels, which could have been linked to its anti-hyperglycemic activity by boosting insulin synthesis from the existing pancreatic β-cells [60].

Recently, it was reported that hyperglycemia and diabetes could be instigated by deficiencies in glycogen synthesis [61]. Correspondingly, our results exhibited a significant diminution in relation to the glycogen content in the diabetic rats, which matched those observed in earlier studies by Oyedemi et al. [62]. These could be explained by a reduction in the ability of the liver to induce glycogen synthase, which augments postprandial glycogen storage, causing a drop in glycogen levels. Sundaram et al. found that insulin promoted hepatic intracellular glycogen deposition by activating glycogen synthase and suppressing the glycogen phosphorylase performances in diabetic rats [63]. Correspondingly, in the current study, the rats doped with the LPE-polyflavonoids manifested a remarkable promotion in their glycogen content compared to the rats treated only with either the hesperidin or diosmin, suggesting a synergistic influence of polyflavonoids on stimulating glycogen synthase. Likewise, previous studies have demonstrated that treatment with only hesperidin modulates insulin secretion, which, in turn, enhances glycogen production [64].

To provide further insights into the activities of liver enzymes, the present results exposed elevated levels of the AST, ALT, and ALP enzymes in the Fructose-STZ-treated rats. These results may be ascribed to hepatic IR, OS, and inflammation associated with excess triglyceride deposition in liver cells, which incite hepatic cell damage and a successive discharge of liver enzymes into the bloodstream [11]. Conversely, the diabetic rats treated with hesperidin, diosmin, and the LPE-polyflavonoids revealed reductions in their liver function enzymes, particularly in the animals treated with the LPE, signifying the hindrance of hepatic impairments, as previously demonstrated [65]. These results agree with the findings from previous reports, which manifested the potency of hesperidin and diosmin in maintaining the integrity of plasma membranes, boosting the restoration of these hepatic enzyme levels [66].

To expand on the performance of other hepatic enzymes, the diabetic group in the current research illustrated noticeable reductions in their activities of HK, GK, and G6Pd. It is well known that HK is a rate-limiting enzyme in the glycolytic process and has a pivotal function in the first phosphorylation of glucose during glycolysis. Moreover, it has a paramount role in the glucose homeostasis in the liver and the progression of DM [67]. Prior investigations have exposed a decrease in the HK enzyme activities in several target tissues during the impaired intake of glucose in diabetic rats [68]. This clearly proposes that the increased blood glucose level could have been correlated with a decrease in the activities of these hepatic enzymes in the diabetic rats [69]. On the other hand, the application of the LPE-polyflavonoids significantly improved HK, GK, and G6Pd compared to those in the hesperidin- and diosmin-treated rats. It has been postulated that the modulating influence of hesperidin in hepatic and extra-hepatic tissues is attributed to the promotion of insulin secretion by existing β-cells [64]. Furthermore, diosmin improved the liver G6PD activity, along with an increase in insulin synthesis. Therefore, the elevation in nicotinamide adenine diphosphate synthesis and reduction in OS could be reported as being due to an enhancement in the glucose influx into the pentose monophosphate shunt [70].

It is worth mentioning that a reduction in albumin levels is not only linked to amplified glucose levels, but also increases vulnerability to DM [71]. Our results thus exhibited a reduction in the albumin concentrations in the diabetic rats. In contrast, the treatment with hesperidin, diosmin, and the LPE-polyflavonoids increased these albumin levels, with the highest level being observed in the case of the polyflavonoids. Hesperidin and diosmin were found to adjust the level of albumin, as a result of their protection against hepatic injury [72,73].

Importantly, the elevations in the cholesterol, TG, and LDL-C levels with a diminution in the HDL-C levels in the diabetic rats in this study indicated insulin deficiency, an excessive mobilization of chylomicrons, and VLDL, leading to hypertriglyceridemia, as has been previously elucidated [74]. On the contrary, the LPE-polyflavonoids treatment ameliorated the entire lipid profile to a greater extent than that of hesperidin and diosmin, stemming from the positive influence of the flavonoid compounds being used together. Prior investigations have accentuated the capacity of hesperidin to preclude cholesterol production and absorption, in addition to the efficacy of diosmin in reducing cholesterol and TG. This is likely related to the effect of flavonoids on inhibiting the activities of both hepatic HMG-CoA reductase and acyl CoA cholesterol acyl-transferase [75].

By studying the oxidative parameters in both the pancreatic and liver tissues, our findings demonstrated disorders in the diabetic rats, identified by elevations in the AGEs and TBARS associated with increases in GSH and CAT, implying an overabundance of free radicals in the tissues, which further provoked OS. Likewise, Yazdi et al. reported that hyperglycemia incited OS and subsequent disruptions in the carbohydrate, protein, and lipid metabolism [76]. Furthermore, Vlassara and Uribarri showed that OS is a dominant cause of impairment in pancreatic β-cells in T2DM, accompanied by a generation of AGEs [77]. Conversely, the treatment with hesperidin, diosmin, and the LPE-polyflavonoids significantly lessened the levels of AGEs and TBARS and evidently improved the GSH and CAT levels in the pancreas and liver. These performances stem from the intrinsic antioxidant properties of flavonoids, which function in tissues either by direct radical scavenging or by boosting the cellular antioxidant defense mechanisms [78,79].

Concerning the inflammatory biomarkers, the marked augmentations of the pancreatic TNF-α, IL-1β, and IL-6 and hepatic NF-κB, TNF-α, IL-1β, IL-6, and AFP in the Fructose-STZ-treated rats could be imputed to excessive glucose concentrations, which triggered oxidative-mechanism-mediated NF-κB, prompting pro-inflammatory cytokine expansion [80]. These cytokines not only instigate NADPH oxidase-generated ROS, but also impair the integrity of pancreatic β-cells. Moreover, an increase in TNF-α has been associated with the activation of NF-κB and NO production, leading to the stimulation of the mitochondrial apoptosis pathways and β-cell dysfunction via the hindering of insulin synthesis [81]. These findings match those observed in earlier investigations, which manifested that the AFP expression was substantially amplified in T2DM throughout the early period of diabetes [82].

In contrast to these findings, the supplementation with hesperidin, diosmin, and the LPE-polyflavonoids outstandingly regulated the production of inflammatory biomarkers and AFP compared to that in the Fructose-STZ-diabetic group, with a particular improvement in the presence of polyflavonoids. Prior investigations have shown that treatment with only diosmin revealed an anti-inflammatory effect by attenuating the TNF-α, NF-κB, and IL-1β production [73].

Furthermore, GLUT2 has a crucial role in glucose transportation inside and outside of the hepatocytes [83]. Our results elucidated that the pancreatic and hepatic levels of GLUT4 and GLUT2 were significantly lowered in the diabetic rats. Similarly, Xu et al. [83] demonstrated that STZ-diabetic animals had diminished levels of hepatic GLUT2 and GLUT4. Thus, an inhibition of the GLUT gene levels is one of the leading causes for hyperglycemia in the diabetic state, as a result of a reduction in glucose uptake [84]. On the contrary, the hesperidin-, diosmin-, and LPE-polyflavonoids-treated groups manifested high levels of GLUT2 and GLUT4 in comparison to the diabetic group. These observations are in accordance with Ali et al. [85], who found that the administration of flavonoids to diabetic rats, including hesperidin and quercetin, improved their insulin sensitivity, modulating their GLUT2 levels, while Hsu et al. [86] reported that diosmin treatment reversed a reduction in GLUT4 expression. Accordingly, they concluded that diosmin serves as a positive modulator of glucose metabolism and an inhibitor of hepatic gluconeogenesis in the insulin-deficient case [86].

In our current results, we found that the gene expressions of p-AKT, PI3K, AMPK, and FOXO1 in the pancreases and PI3K and FOXO1 in the livers of the Fructose-STZ group were significantly downregulated. However, the supplementation of hesperidin, diosmin, and the LPE-polyflavonoids remarkably orchestrated the PI3K, p-AKT, FOXO1, and AMPK gene expressions in comparison to those in the diabetic rats. Similar observations were previously demonstrated by Qiu et al. [87], who reported that diabetic rats exhibited lower gene expressions of PI3K, p-AKT, and FOXO1 in their pancreatic and liver tissues. It is well known that the mechanism of insulin fundamentally relies on the sequential activations and correlated interactions of the PI3K, p-AKT, and FOXO1 signals to lessen the glucose in blood [88]. Furthermore, the PI3K/AKT signaling pathways are specifically implicated in hepatic glucose storage and uptake. Moreover, FOXO1 is a key transcription factor downstream of these insulin signaling pathways. Besides the promotion of PI3K by insulin, nuclear exclusion occurs due to the subsequent phosphorylation of AKT and FOXO1. Kinyua et al. [89] indicated that FOXO1 translocation from the nucleus to the cytoplasm inhibits the expressions of FOXO1 gluconeogenic targets, phosphoenolpyruvate carboxylase (PEPCK), and glucose-6-phosphatase (G-6pase). Considering the pivotal role of AMPK, it has previously been postulated that AMPK in T2DM coordinates the anabolic and catabolic processes, in addition to regulating metabolic homeostasis, which is linked to inflammation and oxidative stress [90].

Intriguingly, the rats treated with hesperidin, diosmin, and the LPE-polyflavonoids showed an upregulation of their PI3K, p-AKT, FOXO1, and AMPK gene expressions. These results may be explained by the capacity of flavonoids and their metabolites to immediately exert an amendment in the cellular system via various signaling pathways or via their interaction with intracellular signaling cascades, such as AMPK and the p-AKT signaling pathways [91]. It can be inferred from these findings that the treatment with the LPE-polyflavonoids showed greater expressions of the previous genes compared to a single flavonoid, implying their potency in orchestrating the expression of the genes responsible for decreasing the glucose level in blood, which mimics the interaction of insulin within cells.

To verify the previous findings, we inspected the histopathological features of the pancreatic and liver tissues harvested from all the studied rat groups. As anticipated, various alterations were perceived in the diabetic rats, such as a degeneration of their pancreatic islets with an irregular outline and vacuolated cytoplasm and other anomalies, which were chiefly related to glucose toxicity and lipotoxicity [92]. Prior investigations have described that diabetic rats exhibit shrinkage in the size of their pancreatic islets and degranulated β-cells, giving rise to hyperglycemia due to an inadequate β-cell mass. These can be elucidated as a consequence of diabetes progression [55]. Conversely, the treatment of the diabetic rats with hesperidin, diosmin, and the LPE-polyflavonoids displayed manifestations of β-cells regeneration and enhancement in the pancreatic tissues, which could be derived from the antioxidant attributes of the flavonoids. Moreover, Ghorbani et al. [93] justified these modifications as being due to the prophylactic mechanisms of flavonoids toward β-cell survival and their capacity to alleviate OS, which eventually thwarts the caspase cascade and DNA damage, protecting pancreatic cells against autophagy, apoptosis, or necrosis.

**Figure 16:**
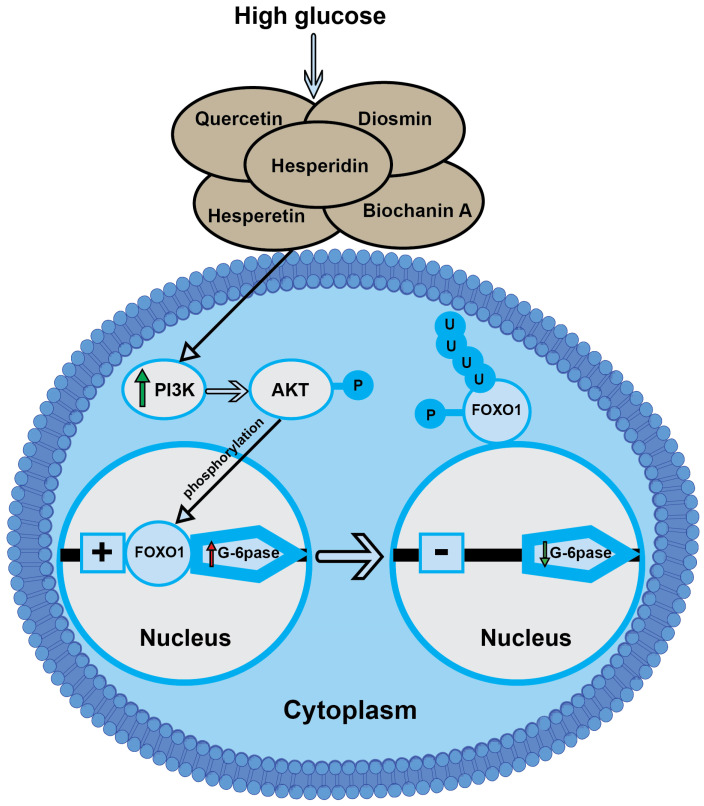
A schematic illustration of the potential mechanism of LPE-polyflavonoids based on the evidence presented in our current study, which demonstrates the respective signaling pathways of LPE-polyflavonoids in mitigating T2DM through diminishing the rates of gluconeogenesis and glycogenolysis. Abbreviations: PI3K: phosphatidylinositol 3-kinase; p-AKT: phospho-Akt or protein kinase B; FOXO1: forkhead box-O; G-6pase: glucose 6-phosphatase; P: phosphorylation; and U: Ubiquitination.

Our histopathological results, in relation to the hepatic tissues in the diabetic rats, were in agreement with those observed by Abdelmageed et al. [5], who provided evidence for the appearance of hepatic and aortic lesions in diabetic rats on account of hyperglycemia, hyperinsulinemia, elevated OS, and hyperlipidemia. Furthermore, we detected a loss in the hepatocyte architecture alongside hepatic necrosis, which is in line with the results of Huang et al. [94]. Moreover, the hepatic steatosis observed in T2DM is likely to be related to a lipid metabolism disorder associated with an abnormal lipid deposition in hepatocytes [95]. Significantly, the rats treated with the LPE-polyflavonoids showed remarkable ameliorations in their hepatic tissues compared to those treated with one of the studied flavonoids. Notably, hesperidin has previously shown a marked improvement in the liver by raising cellular antioxidants [64]. In addition, the diosmin obviously improved the hepatic tissue by restoring its typical structure and architecture, with a centrally positioned vein and undamaged hepatocytes [25]. Considering all of this evidence presented in our study, we could suggest the potential metabolic pathway of LPE-polyflavonoids in attenuating type 2 diabetes mellitus and its deleterious consequences, which mirrors the action of insulin, as portrayed in Figure 16.

## 5. Conclusions

In conclusion, this study highlighted, for the first time, the curative and synergistic influences of the polyflavonoids in lemon peel extract (LPE-polyflavonoids) on governing the glucose level in the bloodstream of diabetic rats and restoring the morphology and activity of pancreatic and hepatic cells, in comparison to diabetic rats treated with a single flavonoid agent. Interestingly, superimposed polyflavonoid structures were observed for hesperidin, quercetin, and hesperetin through the identification of active sites. Furthermore, the docked complex binding affinity supported the robust interaction present in hesperidin (−11.2 kcal/mol). The common key residues in the lipid kinase enzyme PI3K explained the favorable inhibition of the LPE-polyflavonoids. Furthermore, the physicochemical and pharmacokinetic properties were studied and demonstrated significant drug-likeness behavior for hesperidin with a low GI absorption, no BBB parameter effect, and no PAINS alert. Most importantly, the in vivo investigations manifested that the LPE-polyflavonoids revealed an antidiabetic, antioxidant, and anti-inflammatory efficiency by regulating the insulin, glucose, glycogen levels, liver function, and oxidative and inflammatory biomarkers. These properties hindered oxidative stress and its respective complications within the pancreatic and hepatic cells, including inflammatory and cellular impairments. Significantly, the LPE-polyflavonoids manifested a coordination of the PI3K, AKT, AMPK, and FOXO1 signaling pathways in the restoration of the histopathological alterations in the pancreas and liver tissues. Altogether, LPE-polyflavonoids could be implemented to manage diabetes mellitus and its consequences. Therefore, future investigations could focus on loading LPE-polyflavonoids onto different biopolymers with various formulations as drug carriers and delivering them to intended target sites, which could sustain the activity of the extract until its release into the selected site.

## Figures and Tables

**Table 1:** The nucleotide sequences of the primers applied in qRT-PCR.

Gene	Forward Primer	Reverse Primer
PI3K	5′-CCTGGTAACTGCAACACTTC-3′	5′-AACGAATTCAAACCTACCCG-3′
FOXO1	5′-TCATCCAATTGGTCTTGTGG-3′	5′-GTGTTTGCCTGTCTACCTTT-3′
AMPK	5′-TGTGAAGATCGGACACTACG-3′	5′-TAACTGCCACTTTATGGCCT-3′
β-Actin	5′-ATGTGGCTGAGGACTTTGATT-3′	5′-ATCTATGCCGTGGATACTTGG-3′

Accession number: NM_001371300.2, XM_039103268.1, NM_023991.1, and XM_039089807.1, respectively.

**Table 2:** Concentrations of flavonoid compounds in LPE obtained by HPLC analysis.

Flavonoid Compounds	R.T./min	Con. (mg/kg)
Diosmin	22.878	62.75
Biochanin A	17.477	62.35
Hesperidin	6.254	59.18
Quercetin	14.297	28.32
Hesperetin	16.147	9.31

**Table 3:** Pharmacokinetic parameters and drug-likeness properties of the LPE-polyflavonoids.

	Lig I	Lig II	Lig III	Lig IV	Lig V	MYS Control
**Physicochemical properties**						
Molecular weight (Da)	608.54	284.26	610.56	302.24	302.28	318.24
Log P_o/w_ (MLOGP)	−3.23	0.77	−3.04	−0.56	0.41	
Number of H-bond acceptors	15	5	15	7	6	8
Number of H-bond donors	8	2	8	5	3	6
Molar refractivity	143.82	78.46	141.41	78.04	78.06	80.06
Number of rotatable bonds	7	2	7	1	2	1
TPSA (Å^2^)	238.2	79.9	234.29	131.36		151.59
**Pharmacokinetics**						
Gastrointestinal (GI) absorption	Low	High	Low	High	High	Low
Blood–brain barrier (BBB) permeant	No	No	No	No	No	No
P-glycoprotein substrate	Yes	No	Yes	No	Yes	No
Log Kp (skin permeation)	−9.91	−5.91	−10.12	−7.05	−6.3	−7.4
**Drug likeness**						
Log S (ESOL)	−3.51	−3.92	−4.33	−3.16	−3.62	−3.01
Water solubility class	Moderately soluble	Moderately soluble	Moderately soluble	Soluble	Soluble	Soluble
Lipinski rule	No	Yes	No	Yes	Yes	Yes
Ghose	No	Yes	No	Yes	Yes	Yes
Veber	No	Yes	No	Yes	Yes	No
Egan	No	Yes	No	Yes	Yes	No
PAINs	0 alerts	0 alerts	0 alerts	1 alert: catechol_A	0 alerts	1 alert: catechol_A
Bioavailability score	0.17	0.55	0.17	0.55	0.55	0.55
**Metabolism**						
CYP1A2 inhibitor	No	Yes	No	Yes	Yes	Yes
CYP2C19 inhibitor	No	No	No	No	No	No
CYP2C9 inhibitor	No	No	No	No	No	No
CYP2D6 inhibitor	No	Yes	No	Yes	No	No
CYP3A4 inhibitor	No	Yes	No	Yes	Yes	Yes

## Data Availability

The datasets generated during the current study are available from the corresponding authors upon reasonable request.

## Abbreviations

T2DM: Type 2 diabetes mellitus; PI3K: phosphatidylinositol 3-kinase; p-AKT: phospho-Akt or protein kinase B; AMPK: adenosine monophosphate-activated protein kinase; SIRT1: sirtuin; mTOR: mammalian target of rapamycin; FOXO1: forkhead box-O1; GK: glucokinase; HK: hepatic hexokinase; G6Pd: glucose-6-phosphate dehydrogenase; AGEs: advanced glycation end-products; AST: Aspartate aminotransferase; ALT: alanine transaminase; ALP: alkaline phosphatase; CAT: catalase activity; GSH: glutathione; TBARS: thiobarbituric acid reactive substance; TNF- α: tumor necrosis factor alpha; IL-1β: interleukin-1β; IL-6: interleukin-6; NF-κB: nuclear factor kappa B; AFP: alpha-fetoprotein; and MAPK: mitogen activated protein kinases.
